# Twenty-First Annual Report of Middletown State Hospital

**Published:** 1892-03

**Authors:** 


THE TWENTY-FIRST ANNUAL REPORT of the Middletown State Hospital, at Middletown, New York. Transmitted to the Legislature January, 1892. Albany: James B. Lyon, State Printer, 1892.

The purpose of this volume is apparent from its title. The institution itself is designed for the treatment of the insane. The report gives a history of the founding of the institution, and its record for the year ending September 30th, 1891. From the latter part of the statement it appears that 961 patients have been treated in this hospital during the past year.

The most enlightened method of treatment of the insane only is pursued in this hospital, including of course homœopathic medicine. The advanced views of the management in regard to care of the insane are fully set forth in this report. It is indeed a most interesting document. W. M. J.

